# Development of Non-Invasive Biosensors for Neonatal Jaundice Detection: A Review

**DOI:** 10.3390/bios14050254

**Published:** 2024-05-17

**Authors:** Chandan Jyoti Hazarika, Alee Borah, Poly Gogoi, Shrimanta S. Ramchiary, Bethuel Daurai, Manashjit Gogoi, Manob Jyoti Saikia

**Affiliations:** 1 Department of Biomedical Engineering, North-Eastern Hill University, Shillong, Meghalaya 793022, Indiashrimanta.s@gmail.com (S.S.R.);; 2 Department of Electrical Engineering, University of North Florida, Jacksonville, FL 32224, USA

**Keywords:** neonatal jaundice, non-invasive, bilirubin, biosensor, biomarkers

## Abstract

One of the most common problems many babies encounter is neonatal jaundice. The symptoms are yellowing of the skin or eyes because of bilirubin (from above 2.0 to 2.5 mg/dL in the blood). If left untreated, it can lead to serious neurological complications. Traditionally, jaundice detection has relied on invasive blood tests, but developing non-invasive biosensors has provided an alternative approach. This systematic review aims to assess the advancement of these biosensors. This review discusses the many known invasive and non-invasive diagnostic modalities for detecting neonatal jaundice and their limitations. It also notes that the recent research and development on non-invasive biosensors for neonatal jaundice diagnosis is still in its early stages, with the majority of investigations being in vitro or at the pre-clinical level. Non-invasive biosensors could revolutionize neonatal jaundice detection; however, a number of issues still need to be solved before this can happen. These consist of in-depth validation studies, affordable and user-friendly gadgets, and regulatory authority approval. To create biosensors that meet regulatory requirements, additional research is required to make them more precise and affordable.

## 1. Introduction

Jaundice is a medical condition characterized by high bilirubin levels in the human body, which can affect babies, children, and adults. The word “jaundice” is a complicated illness whose name is taken from the French word “jaune,” which means “yellow” in English, and jaundice means yellow disease. It often manifests as jaundice, affecting the skin, mucous membranes, and eyes [1]. Bilirubin, a result of heme degradation, presents risks because of its hazardous nature [2]. The residual amount of bilirubin is derived from the degradation of other heme-containing proteins, such as cytochromes, myoglobin, and peroxidase, predominantly present in the liver and muscles.

Among babies (newborns) worldwide, jaundice is the most frequent clinical condition. A total of 80% of preterm neonates and 65–75% of term infants suffer from jaundice. One out of every two infants globally suffer from neonatal hyperbilirubinemia. Approximately 1.1 million infants worldwide are impacted by acute jaundice annually. The highest incidence of newborn jaundice has been observed in South Asia and Sub-Saharan Africa. Nigeria has a 100-fold higher risk of newborn jaundice than developed countries. The burden of newborn jaundice gradually diminishes as income levels rise from low to middle to high. The number of cases of newborn jaundice per 1000 live births in Africa ranges from 667.8 to 738.5; in South Asia, from 251.3 to 473.2; and in America and Europe, from 4.4 to 3.7, respectively [3,4].

**Figure 1:**
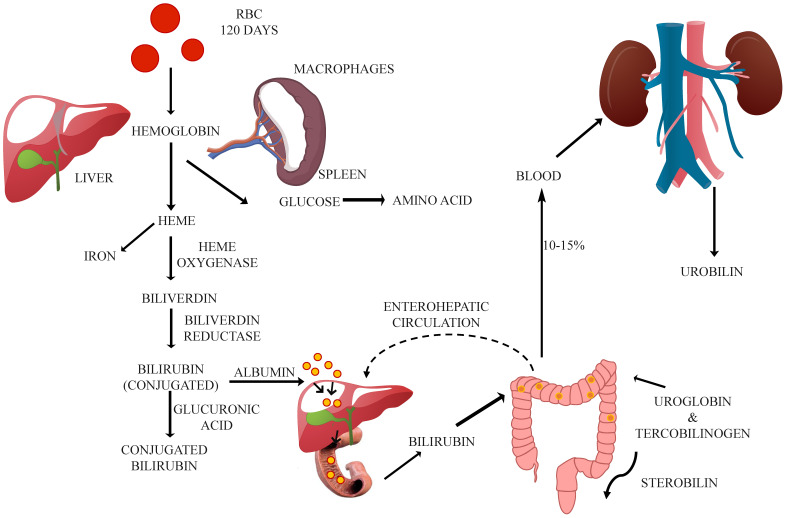
Schematic representation that summarizes the complex process of bilirubin metabolism, from the breakdown of heme to the excretion of bilirubin in bile and urine. It also highlights key enzymes like heme oxygenase, biliverdin reductase, and UGT, which are involved in converting bilirubin into its water-soluble form, bilirubin glucuronides. This image also depicts the transport of bilirubin hepatocytes, where further modification occurs before excretion via the bile ducts.

The formation of bilirubin is summarized as a two-phase process (demonstrated in Figure 1) [2,5,6]. In the first phase, biliverdin is formed from heme (four pyrroles connected by carbon bridges, with an iron atom in the middle). In the second phase, reductase converts biliverdin into water-insoluble unconjugated bilirubin. It is then extracted from the body and combined with albumin to travel through the blood and reach the liver when conjugation occurs [7]. In the liver, conjugate bilirubin (water soluble) is formed due to the addition of glucuronic acid and unconjugated bilirubin by the glucuronyl transferase enzyme [7,8]. Through the biliary system, the conjugate bilirubin then enters the intestine. Most of the conjugated bilirubin is predominantly absorbed by the intestinal cells and later discharged via urine as urobilinogen [9].

The unabsorbed parts of bilirubin are discharged through stool as stercobilinogen. The predominance of conjugated or unconjugated bilirubin results from an alteration of bilirubin metabolism, leading to hyperbilirubinemia or jaundice. The predominance of conjugated bilirubin (20% or more of the body’s total bilirubin at 2 mg/dL) is known as conjugated hyperbilirubinemia. The biological reference of total bilirubin is 0.1–1.2 mg/dL (1.71–20.5 mol/L) and conjugated bilirubin is 0.3 mg/dL or less (5.1 mol/L) [10].

The presence of hyperbilirubinemia in neonates can be either pathological or physiological. During pregnancy, the placenta eliminates bilirubin from the baby’s blood, but after birth, the baby’s liver assumes this function. Newborns produce bilirubin in the range of 6–8 mg/kg/day due to increased red blood cell turnover and relative polycythaemia. Bilirubin production is higher in newborns than adults, gradually decreasing to normal levels within 10–14 days of birth. Babies cannot get rid of much bilirubin in the first few days after birth, because their livers may not be strong enough to remove it on their own (physiological jaundice) [11,12,13,14,15].

The serum bilirubin (total) level may increase to 17 mg/dL (291 mol/L) in cases of physiological jaundice. Premature birth, an elevated bilirubin load due to relative polycythaemia, a reduced erythrocyte life expectancy, immature hepatic absorption, conjugation processes, a decreased volume, and feeding intervals could lead to mild dehydration, and an exaggerated from of physiological jaundice are all risk factors for infants with multiple risk factors. Pathological jaundice, beginning within 24 h after delivery, the start of jaundice, and quickly increasing total blood bilirubin concentrations are some other causes of jaundice [11,12,14,15,16].

High bilirubin levels can have several effects on the body, leading to various complications and conditions. Severe hyperbilirubinemia in newborns can result in bilirubin encephalopathy or kernicterus, which can cause permanent brain damage and neurological impairments. This may manifest symptoms like poor feeding, lethargy, high-pitched crying, muscle rigidity, seizures, and developmental delays. In some cases, untreated bilirubin encephalopathy can contribute to the development of cerebral palsy, a group of movement and coordination disorders. Additionally, high bilirubin levels can cause hearing loss or deafness in newborns by damaging the auditory nerve pathways. Elevated bilirubin levels can also contribute to the formation of gallstones, leading to symptoms like abdominal pain, nausea, and jaundice. Liver dysfunction, indicated by high bilirubin levels, can result from conditions such as hepatitis, cirrhosis, or liver failure, leading to symptoms of jaundice, fatigue, abdominal pain, and changes in appetite. Prompt diagnosis, monitoring, and appropriate treatment are crucial in preventing or managing the potential complications associated with high bilirubin levels [6,17].

Jaundice in newborns is currently diagnosed using a variety of biomarkers, including haptoglobin, albumin, alkaline phosphatase (ALP) (Abbreviations), and bilirubin from the cord blood. The total blood bilirubin level of the newborns and the biomarkers may be compared to determine the risk of developing neonatal jaundice, facilitating the early identification of infant jaundice. The probability of developing neonatal jaundice may be calculated by correlating the biomarkers with the total blood bilirubin level of the infants, aiding early newborn jaundice detection. Nearly all babies have higher bilirubin levels within 1–2 days of birth. Indirect bilirubin levels in newborns should typically be below 5.2 mg/dL within the initial 24 h after birth. Neonatal hyperbilirubinemia is classified when the blood levels of bilirubin are over 5 mg/dL [1].

Albumin is a major binding protein found in human neonates. The fatal albumin has an anodic electrophoretic migration at pH 5.3 and a greater affinity to bind the bromocresol green dye [18]. By competing with tissue for bilirubin binding, albumin decreases bilirubin toxicity. As a result, there are reasons to suspect that albumin levels in cord blood play a decisive role in developing severe jaundice [19,20,21,22]. The biological reference is from 3.5 to 5.5 g/dL [21].

Haptoglobin levels in Umbilical Cord Blood decline during haemolysis due to the binding of free haemoglobin. A normal haptoglobin level ranges from 45 to 200 mg/dL of blood. Studies aims to predict the incidence of jaundice in the future [23,24]. Albumin is a major binding protein found in human neonates. The fatal albumin has an anodic electrophoretic migration at pH 5.3 and a greater affinity to bind the bromocresol green dye. By competing with tissue for bilirubin binding, albumin decreases bilirubin toxicity. As a result, there are reasons to suspect that albumin levels in cord blood significantly impact how severe jaundice develops. The normal range is from 3.5 to 5.5 g/dL [22]. ALP is present in several tissues throughout the human body, with particularly high concentrations in the liver, bile duct, kidney, bone, intestinal mucosa, and placenta [25]. The biological reference of ALP in blood is 20–140 U/L. In children and pregnant women, the ALP level is slightly higher than the normal level [26]. The risk of developing newborn jaundice can be diagnosed by correlating the biomarkers with the total serum bilirubin level of the neonates, aiding in the early detection of newborn jaundice. Correlating the biomarkers with the newborns’ total blood bilirubin level can determine the risk of developing neonatal jaundice, helping detect neonatal jaundice earlier.

The prompt and precise identification of jaundice is crucial for efficient treatment and complication prevention [27]. Biosensors have become valuable tools for quickly, sensitively, and non-invasively detecting bilirubin levels, transforming how infant jaundice screening and treatment are approached [28]. Conventional jaundice diagnosis approaches, like visual evaluation and total serum bilirubin (TSB) (Abbreviations) tests, have drawbacks in terms of precision, invasiveness, and availability, particularly in resource-constrained environments [29]. Biosensors will have benefits such as real-time monitoring, low sample volume needs, and the potential for point-of-care testing, surpassing traditional approaches [30]. Biosensors can help identify at-risk neonates early, allowing for prompt therapies to avert issues related to severe hyperbilirubinemia [31,32].

## 2. Conventional Diagnostic Techniques for Neonatal Jaundice Detection

### 2.1. Visual Assessments

Visual inspections remain the primary method for detecting jaundice in newborns [33]. Healthcare providers assess jaundice based on the coloration of the skin and mucous membranes, primarily focusing on the sclera, face, and trunk. However, the subjective nature of visual assessments poses challenges, leading to variations in interpretation among clinicians [29]. Moreover, factors such as skin tone, lighting conditions, and gestational age can influence the accuracy of visual inspections, necessitating complementary diagnostic tools [34].

**Figure 2:**
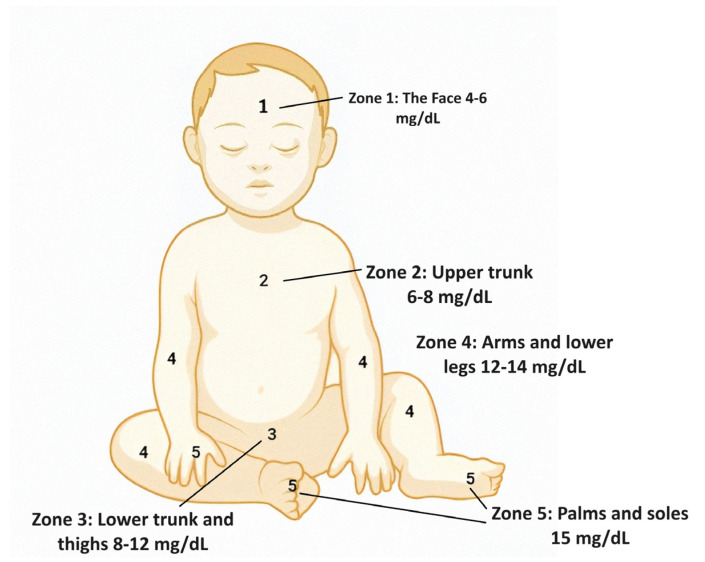
This schematic image illustrates the application of Kramer’s rule, a visual assessment tool, for evaluating the severity of neonatal jaundice. The chart displays a range of skin colour shades, allowing healthcare professionals to match the infant’s skin tone and estimate the corresponding bilirubin level.

In resource-limited areas, visual assessments using Kramer’s method have been widely employed for diagnosing neonatal jaundice [33,35]. This approach involves observing the skin colour and assessing the degree of jaundice. However, conflicting findings exist regarding the reliability of Kramer’s method, especially among neonates of black descent. Based on the cephalocaudal region, Kramer’s method produced a 5-point visual grading system to assess the degree of jaundice (head to extremities) [35,36]. This is the first stage of identifying newborn jaundice. This can be used as a screening tool by trained primary healthcare providers. The different zones for visual assessments include the following (shown in Figure 2):

Zone 1: 4–6 mg/dL;

Zone 2: 6–8 mg/dL;

Zone 3: 8–12 mg/dL;

Zone 4: 12–14 mg/dL;

Zone 5: 15 mg/dL (dangerous jaundice stage).

### 2.2. Total Serum Bilirubin

This is the gold standard method, which involves drawing blood from the infant and measuring bilirubin levels. It also provides accurate results but requires invasive blood collection. Hyperbilirubinemia causes newborn jaundice, one of the most typical illnesses in newborns that affects both preterm and term children [37]. For this reason, different diagnostic methods are used to detect or diagnose jaundice in newborn babies. Jaundice is diagnosed by bilirubin exceeding 2.0–2.5 mg/dL [3,4]. To estimate bilirubin levels, the following methods are used, which determine the severity of neonatal jaundice.

#### 2.2.1. The Diazo Method

In the diazo method, a reaction between diazotized sulfanilic acid and bilirubin results in formation (a coloured compound), which is determined to estimate the amount of bilirubin in patients. There are numerous ways for determining bilirubin, the majority of which follow the diazo method described by Hijmans van den Bergh [38,39]. Estimating bilirubin levels using the diazo technique is inaccurate, particularly in identifying low amounts of bilirubin. It has limitations such as the interference from haemolysis and lipemia or paraproteins, which can be especially problematic in newborns and infant populations. In the case of the peroxide diazo method, an aliquot of the substance (about 25 mL) is to be blended with a minor amount of HRP (horseradish peroxidase) and peroxide. By inactivating the horseradish peroxidase, the sulfanilic acid stops the oxidation of bilirubin. Only unoxidized bilirubin can yield diazo derivatives, since the by-products of bilirubin oxidation are diazo negative. The amount of horseradish peroxidase determines the diluted sample used to assess the unbound bilirubin and peroxidase added. However, this approach is applicable only when the majority of the total bilirubin is unconjugated bilirubin without modifications.

#### 2.2.2. High Pressure Liquid Chromatography

One of the most popular methods for the quick separation and more accurate measurement of the four bilirubin components from the sample serum is HPLC (Abbreviations), which includes unconjugated bilirubin, mono-conjugated bilirubin, di-conjugated bilirubin, and a portion permanently bound to a protein (α-bilirubin, β-bilirubin, γ-bilirubin, and δ-bilirubin, respectively) [28,40]. The processing time for these two techniques is typically around 30 min. This approach allows for the rapid separation and simultaneous identification of bilirubin fractions in serum and is more sensitive than the traditional diazo technique [38,39,41]. Although invasive strategies provide maximum accuracy, they can be complicated by contamination if sterilization methods are inadequate. Furthermore, repeated blood draws required for continuous monitoring can lead to anaemia, pain, and distress, causing significant discomfort to the individual [42].

#### 2.2.3. The Enzyme-Based Method

Bilirubin oxidase (BOD) is essential in enzymatic biosensing devices created to measure bilirubin levels [43]. BOD functions as a catalyst in enzymatic biosensing by facilitating the oxidation of bilirubin, resulting in the generation of hydrogen peroxide (H_2_O_2_) [38,39,44]. This enzymatic process is the foundation of the detecting method. The hydrogen peroxide produced acts as a redox indicator, and its electrochemical characteristics are used to measure the amount of bilirubin in a sample.

An established method involves linking the enzymatic process facilitated by BOD with the function of horseradish peroxidase (HRP) (Abbreviations) [43,45]. HRP is an enzyme that interacts with hydrogen peroxide to generate an electrochemical signal. Combining the catalytic activities of BOD and HRP can improve the detection sensitivity and specificity.

#### 2.2.4. Fluorescence Spectroscopy

In an aqueous solution, bilirubin has a comparatively low fluorescence quantum yield. The bound fraction of bilirubin may be determined using fluorescence assays, as bilirubin is more efficient when coupled to its native carrier, albumin, in aqueous solutions (the bilirubin-albumin mixture fluoresces at 520 nm) [46]. The fluorescence analysis of albumin-bound bilirubin can be used to create the binding capacity calculation. 

### 2.3. Transcutaneous Bilirubinometer

This is a non-invasive method which measures bilirubin levels by shining light through the skin by the principle of spectroscopy. This spectrophotometer assay measures the amount of extravascular bilirubin in the skin and subcutaneous tissues, which is related to the commonly used blood bilirubin test. The technique relies on measuring the absorbance of bilirubin at 454 nm, while haemoglobin absorbs light equally at both 454 nm and 528 nm. Haemolysis interference is eliminated by subtracting the absorbance at 528 nm from that at 454 nm, yielding a value mainly attributed to bilirubin [38,39]. It correlates with TSB levels but may have limitations like poor sensitivity when it is used to assess blood bilirubin levels above 250 mol/L and skin colour. These factors can lead to overestimations. The advantages of TcB (Abbreviations) are its non-invasiveness, quick results, cost-effectiveness, and painlessness.

Several TcB devices are available, including the Drager Jaundice meter JM-103, BiliCheck, and Minolta JM-103 [47]. Research has compared these devices in preterm neonates, highlighting their accuracy and reliability. A study by Gothwal et al. found that TcB measurements taken from the covered skin area of jaundiced preterm newborns undergoing phototherapy did not show a correlation with TSB levels and, therefore, cannot be utilised as a substitute for serum bilirubin testing [48].

## 3. Biosensors for the Detection of Neonatal Jaundice

Biosensors are based on a hybrid of physical and chemical sensing techniques. According to the International Union of Pure and Applied Chemistry, a biosensor refers to the analytical devices that transform replication into electrical signals by sensing chemicals that have a highly specific, liberated pH and temperature-like physical parameters [49,50]. The transducer, amplifier, detector, and bio-recognition element are the four essential parts of a biosensor. Chemical substances comprising enzymes, cells, DNA (Abbreviations), proteins, tissues, organelles, antibodies, aptamers, and other components comprise biorecognition elements. A transducer transforms bio-recognition signals into a signal that can be detected. The amplifier increases the signal’s amplitude. The detector analyses the signal to extract useful data [51,52].

**Figure 3:**
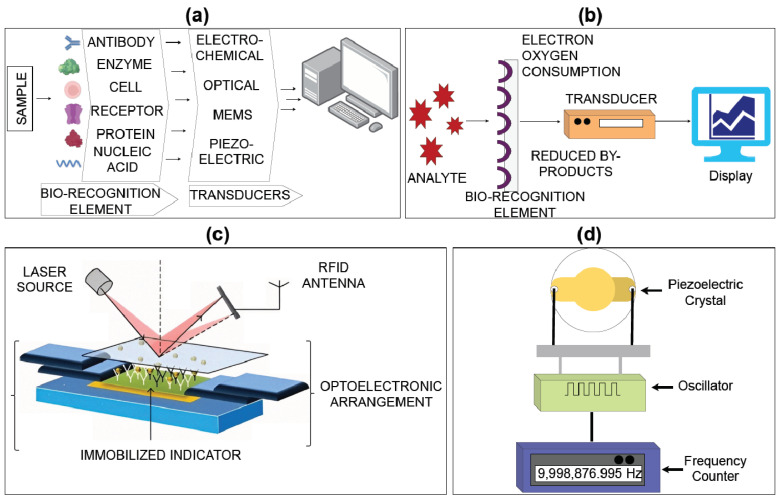
Different types of biosensors and their working principle: (**a**) architecture of biosensors; (**b**) electrochemical biosensor [53]; (**c**) architecture of optical biosensors [54]; (**d**) architecture of piezoelectric biosensor [49].

Figure 3a displays a schematic of a biosensor designed for the precise detection and analysis of target molecules. It shows the essential parts of a biosensor: a recognition element, a transducer, and a signal output. The recognition element, typically a biomolecule or receptor, specifically attaches to the desired target molecule. The transducer transforms the binding event into a detectable signal, which can be visual, electrical, or electrochemical. The signal output offers precise data regarding the existence and amount of the target molecule.

Sensing elements and transducer modes are the two components of biosensors. The sensing components are enzyme-based biosensors that rely on biological recognition and the need to be stable and accessible to catalyse a specified biological response under typical operating conditions. An immune sensor is a biosensor in which antibodies catch the specific target antigen and is established on the fact that antibodies highly attract antigens. A DNA biosensor detaches and relies on the nature of single DNA or immune-response antigen bonds. Cell biosensors are used to employ living cells, and they are dependent on the capacity of living cells for perceiving the intracellular and extracellular microenvironment condition [52,55].

The applications of these biosensors for identifying neonatal jaundice are covered in the following sections. The biosensors reviewed in this report have been categorized according to the types of transducers used.

### 3.1. Invasive Biosensors

#### 3.1.1. Electrochemical Biosensors

Electrochemical biosensors employ biological components, such as an enzyme or an antibody, to find certain analytes in a sample, like bilirubin. When the analytes connect with the biological elements, they create a signal that the electrode can detect. The biological element is coupled to an electrode [52,56,57].

The schematic representation of an electrochemical biosensor is shown in Figure 3b. This image illustrates the essential components of an electrochemical biosensor. An electrochemical biosensor operates based on the measurable electrical properties of the solution, including the electric potential, which is affected by the exchange of electrons or ions during a chemical reaction involving the biomolecule being tested and the target analytes. Biosensors can utilize amperometric, conductometric, or potentiometric transducers to convert chemical information into an identifiable amperometric signal [56].

A novel method was developed by Anzar et al. for detecting bilirubin, which involved utilizing paper-based screen-printed carbon electrodes that are covalently attached to nanoparticles [58]. The nanomaterial used was a silver nanoparticle (AgNP) (Abbreviations). The immobilization of bilirubin oxidase was conducted on the AgNP electrodes. The biosensor’s analytical response was assessed with a potentiostat through cyclic voltammetry (CV) and linear sweep voltammetry (LSV). The paper-based sensor demonstrated superior performance with a decreased Limit of Detection (LOD) (Abbreviations) of 1 g/mL and a linear range of 1–9 mg/mL for bilirubin. Tests for bilirubin in simulated blood serum are utilized to verify its viability.

In another study, Thangamuthu et al. developed a unique method for the selective measurement of bilirubin using screen-printed carbon electrodes, which were created using nanoparticles [59]. Multi-walled carbon nanotubes (MWCNTs) (Abbreviations) and graphene were independently placed on the screen-printed carbon electrodes. The electrocatalytic activity for the biliverdin was detected at +0.25 Volt. Similar to the first peak, a second peak at +0.48 Volt was seen, which indicated that biliverdin had electrochemically changed into purpurin. The graphene variant has a three-fold LOD than MWCNTs and a twice-as-good sensitivity. Bilirubin measurements in blood serum samples were used to verify viability, and an ionic Nafion membrane blocked common biological substrates that interfere to guarantee selectivity. 

In another paper-based biosensor, Bell et al. developed a screen-printed carbon electrode, a novel technique for the estimation of bilirubin. The electrodes were made of a AgNP, which immobilized with bilirubin oxidase, forming a covalent bond. The biosensors’ analytical response was assessed using cyclic and linear sweep voltammetry. The sensor was evaluated using simulated blood serum and had a power LOD of 1 g/mL and a larger linear range of 1–9 g/mL for bilirubin [58,60].

Amperometric biosensors, a type of electrochemical biosensor, are integrated devices that measure the current produced when an electroactive biological element undergoes oxidation or reduction, providing accurate quantitative data [61,62]. It primarily bases its measurement of the associated level of analytes in the biological sample on the amount of current generated by the analytes through oxidation and reduction reactions [56,57]. Types of amperometric biosensors include enzymatic amperometric biosensors and non-enzymatic amperometric biosensors. 

An advanced amperometric sensor was developed by immobilizing bilirubin oxidase on zirconia-coated silica nanoparticles/a chitosan composite [57,63]. This composite was electrically deposited onto a Au electrode in the sensor. The biosensor features a wide linear range spanning from 0.02 to 50 M and high sensitivity, with a detection limit of 0.1 nM. The biosensor was utilized 150 times in 120 days and showed a significant correlation with a commonly used colorimetric approach for measuring bilirubin levels.

In yet another paper-based sensor, unbound ionic bilirubin in blood was determined by utilizing a polymeric ion-selective membrane [60]. This sensor detects anions such chloride, phosphate, pyruvate, deoxycholate, and lactate. Only 15 µL of serum is needed for analysis by the sensor and could easily measure ionic bilirubin. It also achieved a linear response from 1.0 mM to 0.10 µM. The polymeric ion-selective membrane was cut into a circle and fused to a poly vinyl chloride tube with a AgCl-coated Ag wire inside. Before the analysis, the electrode was submerged in a solution of 10 µM of bilirubin for 3 h for allowing Cl- in the ion-selective membrane to exchange with ionic bilirubin.

#### 3.1.2. Optical Biosensors

An optical biosensor utilizes light to identify biological molecules or chemical compounds. Different optical techniques, such surface plasmon resonance, fluorescence, or interferometry, can be used to detect and analyse this shift. Figure 3c displays the design of an optical biosensor used for detecting target molecules without the need for labels, with high sensitivity. This graphic shows the essential elements of an optical biosensor: a light source, an optical waveguide, a bioreceptor, and a detector. The light source emits light that is directed through the optical waveguide. The bioreceptor, fixed on the waveguide surface, specifically captures the desired target molecule. The binding event causes alterations in optical properties like the refractive index or fluorescence, which are then identified by the detector. 

Li et al. studied an optical method for measuring bilirubin using an enzyme-based fibre-optic fluorescence biosensor [64]. Bilirubin is converted to biliverdin by the action of bilirubin oxidase. The enzymatic reaction consumed oxygen, and, as a result, there was a change in the fluorescence intensity. The LOD was 4.4 × 10^−7^ M. The calibration curve’s linearity ranged from 4.4 × 10^−7^ M to 3.0 × 10^−4^ M.

In a recent study of optical biosensors by Mohamad and Manap, an optical fibre sensor was designed to detect bilirubin content by the absorption of bilirubin in the Ultraviolet/Visible region [57,65]. The Beer–Lambert Law was employed. An empty cuvette was initially used to quantify the incidence intensity as light passed through it. A cuvette was filled with a bilirubin sample before measuring the transmitted intensity. Bilirubin’s theoretical absorbance peaks between 400 nm and 600 nm, similar to what was observed in the study.

#### 3.1.3. Piezoelectric Biosensors 

Piezoelectric biosensors are also called acoustic biosensors, constructed of piezoelectric crystals, which vibrate at certain frequencies and have positive and negative charges [53,66]. Acoustics, or these sound vibrations, are the foundation of piezoelectric biosensors. The resonance frequencies at which piezoelectric crystals vibrate alter when other molecules are deposited on their surfaces. Electronic tools are used to measure these changes. Nevertheless, these crystals cannot oscillate correctly in viscous liquids. Hence, employing piezoelectric biosensors to identify chemicals in a solution is challenging [56]. Figure 3d displays a diagram of a piezoelectric biosensor designed for the precise detection and analysis of target molecules with great sensitivity. This image illustrates the essential components of a piezoelectric biosensor, including piezoelectric material, a bioreceptor, and a detection system. The molecule of interest interacts with the bioreceptor that is fixed on the surface of the piezoelectric material. This contact causes a change in mass or surface tension, leading to a detectable alteration in the resonant frequency.

In a study by Yang and Zhang, surface imprinting was combined with the sol–gel technique to develop a molecular imprint of bilirubin on the hydroxyapatite layer, which was coated on a quartz crystal [57,67]. The frequency change of this piezoelectric biosensor revealed that it has a vast linear range of 0.05–80 µM, a low LOD of 0.01 µM, and a quick reaction time (37 min). This bilirubin biosensor can identify bilirubin in a serum sample.

There was also another study by Yang et al. where a piezoelectric biosensor was developed [57,68,69]. Titanium film was imprinted on by bilirubin which was coated on the quartz crystals. The response time was 30 min, and the LOD was 0.05 µM. According to the evaluation results, the biosensor offered a wide linear range of 0.1–50 µM. This showed an applicability of bilirubin estimation in blood serum.

### 3.2. Non-Invasive Biosensors

Non-invasive biosensors provide a significant benefit in neonatal diagnostics, transforming healthcare for babies. By avoiding invasive treatments, such as blood draws, they reduce discomfort and risk for these delicate individuals. In a study by Resmi et al., a screen-printed carbon electrode (SPCE) was developed to determine the electrochemical oxidation of conjugated bilirubin [70]. The oxidation of bilirubin occurred on SPCE at a lower anodic potential and was highly selective towards conjugated bilirubin in the presence of interfering molecules. The SPCE exhibited a dynamic detention range of 1–600 µM. It was tested with urine samples spiked with bilirubin, which demonstrated a satisfactory response.

#### Optical Biosensors

In a study by Inamori et al., a bromocresol green biosensor was used to measure bilirubin levels on the skin by detecting changes in blue and green light absorption [71]. It also calculates oxygen saturation and heart rate red and infrared light-emitting diode lights (LEDs) (Abbreviations). The device utilizes a Polydimethylsiloxane lens and a silicon interface to enhance the accuracy of bilirubin measurements and to monitor vital signs. The micro control unit controls LED emissions, voltages, and the photodiode signal. This signal is amplified and is converted to digital. The measured data are sent through Bluetooth to a smartphone.

In a study of digital imaging, Hashim et al. developed a technology that identifies jaundice and assesses the need for therapy in newborns [72]. Furthermore, the proposed system possesses the capability to transmit the diagnostic findings to the mobile device of the healthcare professional. The pictures were processed using several techniques, including colour space transformation, to detect the neonate’s skin and define the region of interest automatically. The proposed approach effectively determines whether the newborn is afflicted with jaundice or not. These advantages include its efficacy in detecting jaundice at a TSB level of 14 mg/dL and higher, a swift detection time of only 1 s, and its suitability for use in hospitals and medical centres lacking laboratory facilities and trained medical personnel, owing to its affordability.

In another study on image processing for neonatal detection, Althnian et al. investigated the effectiveness of transfer learning using the skin, eye, and a fusion of skin and eye features [73]. The transfer learning was tested against traditional machine learning models, including a multi-layer perceptron (MLP), a support vector machine (SVM), a decision tree (DT), and random forest (RF). Their results show that transfer learning models performed best with skin features and regular machine learning models with eye features. Traditional MLP, SVM, and RF models performed similarly to eye characteristics and better than DT.

In yet another digital image-based study by Abdulrazzak et al., an extreme gradient boost (XGBoost) machine learning technique was proposed [74]. A USB-connected webcam was used to develop an application for jaundice detection that detects neonates in different positions and lighting conditions in real-time without invasive tests. The technique retrieved skin colour intensities from 767 infant photographs and was tested and validated with machine-learning models. The XGBoost algorithm detected 10 of 10 NICU newborns with 99.63% accuracy in a short period. Using MATLAB, Khanam et al. also designed an automatic region-of-interest (ROI) selection technique, including face detection, face landmark detection, and ROI, i.e., forehead selection [75]. Then, the skin colour of the selected forehead region was analysed in both RGB (Red, Green, and Blue) and YC_b_C_r_ (Luminance, Chrominance) colour spaces. Finally, using a machine-learning algorithm based on RF, they detected jaundice. This also showed a possible application for neonatal jaundice detection (Table 1).

## 4. Challenges and Future Scope

In order to diagnose and monitor newborn jaundice, biosensors have demonstrated significant promise as a non-invasive and effective technique. A few issues still need to be resolved to increase the precision and potential of biosensors for this application. One major challenge is the interference from other substances in the blood, which can affect the specificity and sensitivity of the biosensor. The precision of the bilirubin measurement may be impacted, for instance, by high blood levels of lipids, proteins, or other pigments. Another challenge is the need for a reliable reference standard for bilirubin measurement. Blood sample analysis is the gold standard for determining blood bilirubin levels, although it can be intrusive and time-consuming. There is a need to develop more reliable and accurate reference standards that can be used to validate biosensor measurements.

Additionally, biosensors for neonatal jaundice detection must be designed to be easy to use, affordable, and portable. They should be able to provide real-time results and be suitable for biosensors in neonatal jaundice detection. Advances in sensor technology, such as developing new materials and detection methods, could improve the accuracy and specificity of biosensors. The integration of biosensors with digital health technologies could also improve the efficiency and accessibility of neonatal jaundice detection and monitoring. Overall, developing and refining biosensors for neonatal jaundice detection is an essential area of research with significant potential to improve neonatal health outcomes.

## 5. Conclusions

Biosensors can completely transform how neonatal jaundice is diagnosed and treated. The development of biosensors for this purpose has already shown promising results in terms of accuracy, speed, and cost-effectiveness. Bilirubin, the primary marker of newborn jaundice, has been designed and tested to be detected by various biosensors, including optical, electrochemical, and colorimetric ones. Among these, optical biosensors, such as fluorescence- and absorbance-based biosensors, have shown the most promising results regarding sensitivity and accuracy.

However, some challenges still need to be addressed before biosensors for neonatal jaundice can be widely adopted. These include the standardization of biosensor measurements, the validation of biosensors in clinical settings, and the development of biosensors that can detect other biomarkers besides bilirubin. Overall, biosensors have the potential to provide a rapid, accurate, and cost-effective technique for the diagnosis and management of newborn jaundice, which can significantly improve the outcomes for newborns. 

## Figures and Tables

**Table 1:** Biosensors used for the detection of neonatal jaundice.

Sr. No.	Method	Types of Biosensors	Biomarker	Limit of Detection(LOD)	Reference
1.	Invasive	Electrochemical	Bilirubin	1 µg/mL	[58]
2.	Invasive	Electrochemical	Bilirubin		[59]
3.	Invasive	Optical	Bilirubin	4.4 × 10^−7^ M	[64]
4.	Invasive	Optical	Bilirubin	--	[57]
5.	Invasive	Paper-based	Bilirubin	1 g/mL	[58]
6.	Invasive	Amperometric	Bilirubin	0.1 nM	[63]
7.	Invasive	Potentiometric	Serum bilirubin	15 μL	[60]
8.	Invasive	Piezoelectric	Serum bilirubin	0.01 μM	[57]
9.	Invasive	Piezoelectric	Serum bilirubin	0.05 μM	[57]
10.	Invasive	Amperometric	Bilirubin	4 × 10^−6^ M	[45]
11.	Invasive	Amperometric	Bilirubin	--	[76]
12.	Invasive	Luminescence sensor	Bilirubin	1.75 μM	[77]
13.	Invasive	Fibre optic	Bilirubin	1 × 10^−7^ M	[75]
14.	Invasive	Oxygen amperometric	Bilirubin	6 μM	[76]
15.	Non-invasive	Colorimetric	Transcutaneous bilirubin	--	[71]
16.	Non-invasive	Electrochemical	Bilirubin	1 μM	[70]
17.	Non-invasive	Computer vision system	Skin colour	14 mg/dL	[72]
18.	Non-invasive	Smartphone camera using transfer learning	Skin colour	--	[73]
19.	Non-invasive	USB webcam with machine learning technique	Skin colour	--	[74]
20.	Non-invasive	Frontal image detection based on machine learing	Skin colour	--	[75]

## Abbreviations

ALP	Alkaline phosphatase
HRP	Horseradish peroxidase
HPLC	High-pressure liquid chromatography
TSB	Total serum bilirubin
MWCNT	Multi-walled carbon nanotubes
TcB	Transcutaneous bilirubinometer
DNA	Deoxyribonucleic acid
AgNP	Silver nanoparticle
LOD	Limit of detection
LED	Light-emitting diode
